# The Use of Calf Lymph in Epidemic Small-Pox

**Published:** 1901-03-09

**Authors:** 


### THE USE OF CALF LYMPH IN EPIDEMIC SMALL-POX.

One of the great advantages possessed by the glycerinated calf lymph now used for vaccination over the older method of direct vaccination from arm to arm—the facility, namely, with which large quantities of it can be quickly prepared when required for purposes of revaccination in case of threatened epidemics—is well illustrated by the events which have lately taken place in Glasgow, where small-pox has assumed epidemic proportions. Many hundreds of cases have occurred, and as a not unnatural result a great demand for revaccination has arisen—a demand so great as to have already led to the performance of the operation in nearly a quarter of a million of cases since the outbreak began. It would clearly have been quite impossible to have effected anything approaching such an extensive revaccination by the older methods from arm to arm, and even with tubes it would have been very difficult to obtain a sufficiently large supply of human lymph of a good quality. With calf lymph, however, all is plain sailing.

